# *NSMCE2 / MMS21* structural variant causes chromosome breakage syndrome with primordial dwarfism and severe lung disease

**DOI:** 10.1038/s41431-026-02222-7

**Published:** 2026-09-21

**Authors:** Tomer Poleg, Noam Hadar, Vadim Dolgin, Ginat Narkis, Monica Neuhaus, Ilana Aminov, Ekaterina Eremenko, Amit Safran, Nadav Agam, Matan M. Jean, Ofek Freund, Uriel Wachsman, Bibi Kanengisser-Pines, Lidia Osyntsov, Esther Manor, Ohad S. Birk

**Affiliations:** 1 The Morris Kahn Laboratory of Human Genetics at the National Institute of Biotechnology in the Negev, Ben-Gurion University of the Negev, Beer Sheva, Israel; 2 Faculty of Health Sciences, Ben-Gurion University of the Negev, Beer Sheva, Israel; 3 Genetics Institute, Soroka University Medical Center, Beer-Sheva, Israel; 4 Department of Medical Imaging, Soroka University Medical Center, Beer-Sheva, Israel; 5 Institute of Pathology, Soroka Medical Center, Beer-Sheva, Israel; 6 The Danek Gertner Institute of Human Genetics, Sheba Medical Center, Tel-Hashomer, Ramat Gan, Israel

**Keywords:** Disease genetics, Genetics research

## Abstract

Primordial dwarfism (PD) is a genetic disorder characterized by severe intrauterine and postnatal growth failure. While some subtypes involve impaired DNA damage responses, the molecular basis of PD remains incompletely defined. We describe two siblings of non-consanguineous Indian-Jewish ancestry with a likely autosomal recessive syndrome featuring borderline intrauterine growth restriction, severe postnatal growth restriction, hypotonia, consistent dysmorphic features (elongated face, low-set ears, hypotelorism, prominent nose), hypothyroidism, late-onset insulin-dependent diabetes, borderline immune dysregulation, and recurrent pneumonia with interstitial lung disease, leading to death in adolescence. Karyotype and microarrays were normal, but cytogenetic analysis of PHA-stimulated lymphocytes revealed spontaneous chromosomal breakage and polyploidy, without hypersensitivity to cross-linking agents. Linkage analysis, whole genome sequencing, and Manta structural variant calling identified a homozygous tandem duplication within *NSMCE2*, NC_000008.11(NM_173685.4:c.265-11431_418+8879dup), validated by PCR and Sanger sequencing. RT-PCR of lymphoblastoid cells confirmed an aberrant transcript, predicted to cause a frameshift, premature stop codon, and loss of the SUMO domain. *NSMCE2* encodes a core component of the SMC5/6 complex, essential for chromosome structure and repair. Only one prior report has linked *NSMCE2* mutations to PD. Our findings determine the clinical and molecular spectrum of this severe chromosome breakage syndrome with PD, lung fibrosis, immune-endocrine abnormalities, and early lethality.

## Introduction

Primordial dwarfism (PD) is a rare condition characterized by severe growth impairment evident in the developing fetus. While the initial clinical manifestation of PD is often intra-uterine growth restriction (IUGR), it persists throughout postnatal development, resulting in a substantially diminished adult stature. Sub-types of PD include Seckel syndrome (SS), Meier-Gorlin syndrome (MGS), Microcephalic Osteodysplastic Primordial Dwarfism (MOPD) types I/II/III, and Silver-Russell syndrome (SRS). A defining feature of PD sub-types, excluding SRS, is microcephaly, with small head size relative to the body, commonly known as “Microcephalic Primordial Dwarfism” (MPD) [1, 2] .

In recent years, PD has been classified as a group of single-gene disorders, with more than 10 distinct genes associated with the condition, as documented in Online Mendelian Inheritance in Man (OMIM) [3]. The underlying genetic defects involve a variety of fundamental processes, including genomic replication, centrosome functions, DNA damage response, and DNA repair pathways. Collectively, PD-associated genes encode proteins that facilitate the precise chromosomal segregation and mitotic division of cells [4].

We now define a disease-causing structural variant (SV) within *NSMCE2*, encoding a protein of the Structural Maintenance of Chromosomes SMC5/6 complex, a crucial player in the organization and dynamics of chromosome structures across all living organisms [5]. We delineate the disease phenotype and summarize the scarce literature to date regarding this rare syndrome.

## Methods

### Clinical phenotyping

The study was approved by the Soroka Medical Center Institutional Review Board (IRB; approval #5071 G) and the Israel Ministry of Health National Helsinki Committee (approval #920100319). Written informed consent was obtained from all individuals studied or their legal guardians. Phenotyping was done by senior clinical geneticist, pediatric endocrinologist, and clinical cytogeneticist.

### Genetic analysis

Cytogenetic studies of peripheral blood cells, as well as PHA-stimulation and testing the effects of diepoxybutane (DEB) and mitomycin C (MMC), were done using standard procedures as previously described [6]. For molecular studies, genomic DNA was extracted from peripheral blood leukocytes using the E.Z.N.A Blood DNA kit (Omega Bio-tek, Nor-cross, GA, USA) as previously described [7]. Genome-wide linkage analysis of all affected individuals (II-1, II-2) and four healthy controls (I-1,2, II-3,4) was performed using Illumina Omni Express Beadchip with more than 750 K SNP loci per sample (Illumina Inc.). Homozygosity mapping analysis was conducted utilizing the Homozygosity-Mapper tool [8] (http://www.homozygositymapper.org/). Genomic analyses, encompassing sequencing, read alignment, variant calling, and subsequent annotation, were outsourced to Macrogen Europe B.V. (Amsterdam, The Netherlands). For WES, libraries were prepared using the Agilent SureSelect Exome V7 kit (post-capture) and sequenced on an Illumina NovaSeq 6000 platform with a 150-bp paired-end configuration. WGS was similarly conducted on the Illumina NovaSeq 6000 utilizing a TruSeq PCR-free library preparation protocol (350-bp insert size), yielding approximately 110 Gb of raw data. Raw data reads were aligned to GRCh38 reference genome using BWA-MEM and Picard. Variant calling was done using GATK 4.3.0.0, and Manta [9] 1.6.0. Data were analyzed using VARista [10] software and Genoox analysis software, Franklin (https://franklin.genoox.com/clinical-db/home). Variant nomenclature was validated using the VariantValidator tool (https://variantvalidator.org) in accordance with HGVS guidelines [11, 12]. The identified variant was submitted to the ClinVar database (accession number SUB16270621). Segregation analysis of the structural variant within the studied kindred was done through PCR amplification and gel electrophoresis using primers flanking the junction point of the duplicated regions: Forward 5′-CCTCTGCTCAGGCTATTGTACTTG-3′ Reverse 5′- CGAAGTGGGACAGATGACGG-3′. (628 bp amplicon; annealing temperature 66.1°C; extension time 38 s). Sanger sequencing of the amplicon confirmed the precise junction point between the two duplicated regions.

### PHA-stimulated lymphocyte assay

peripheral blood lymphocytes were cultured in the presence of phytohemagglutinin (PHA) using standard cytogenetic protocols, followed by metaphase arrest, hypotonic treatment, fixation, slide preparation, and chromosomal analysis.

### Reverse transcription and real-time quantitative PCR

Epstein–Barr virus (EBV)-transformed lymphoblastoid cell lines were generated as previously described [13]. Total RNA was extracted from EBV-transformed cells of two patients (II-1, II-2) and two WT controls using the GENzol™ Tri RNA Pure Kit (Geneaid Biotech Ltd.). Single-stranded cDNA libraries were prepared using Verso cDNA synthesis Kit (Thermo Scientific). The following primer sequences were utilized for amplifying *NSMCE2* cDNA: Forward primer 5’-GAGCAAGAATTCTGATGCAGAC-3’ and Reverse primer 5’-CTGGACGTTCTTCTTTCAC-3’, resulting in a 102 bp amplicon of the mutated allele, which includes the junction site between the two TDs. Quantitative Real-time PCR (qRT-PCR) was done using a Hy-SYBR Power mix (hylabs). *NSMCE2* transcript levels were normalized to Glyceraldehyde 3-phosphate dehydrogenase (*GAPDH*) housekeeping gene. PCR conditions: annealing temperature 62^o^C, extension time 20 s, 40 cycles. Differences between groups were assessed using an exact two-tailed Mann-Whitney test. A *P* value < 0.05 was considered statistically significant.

## Results

### Clinical findings

**Fig. 1:**
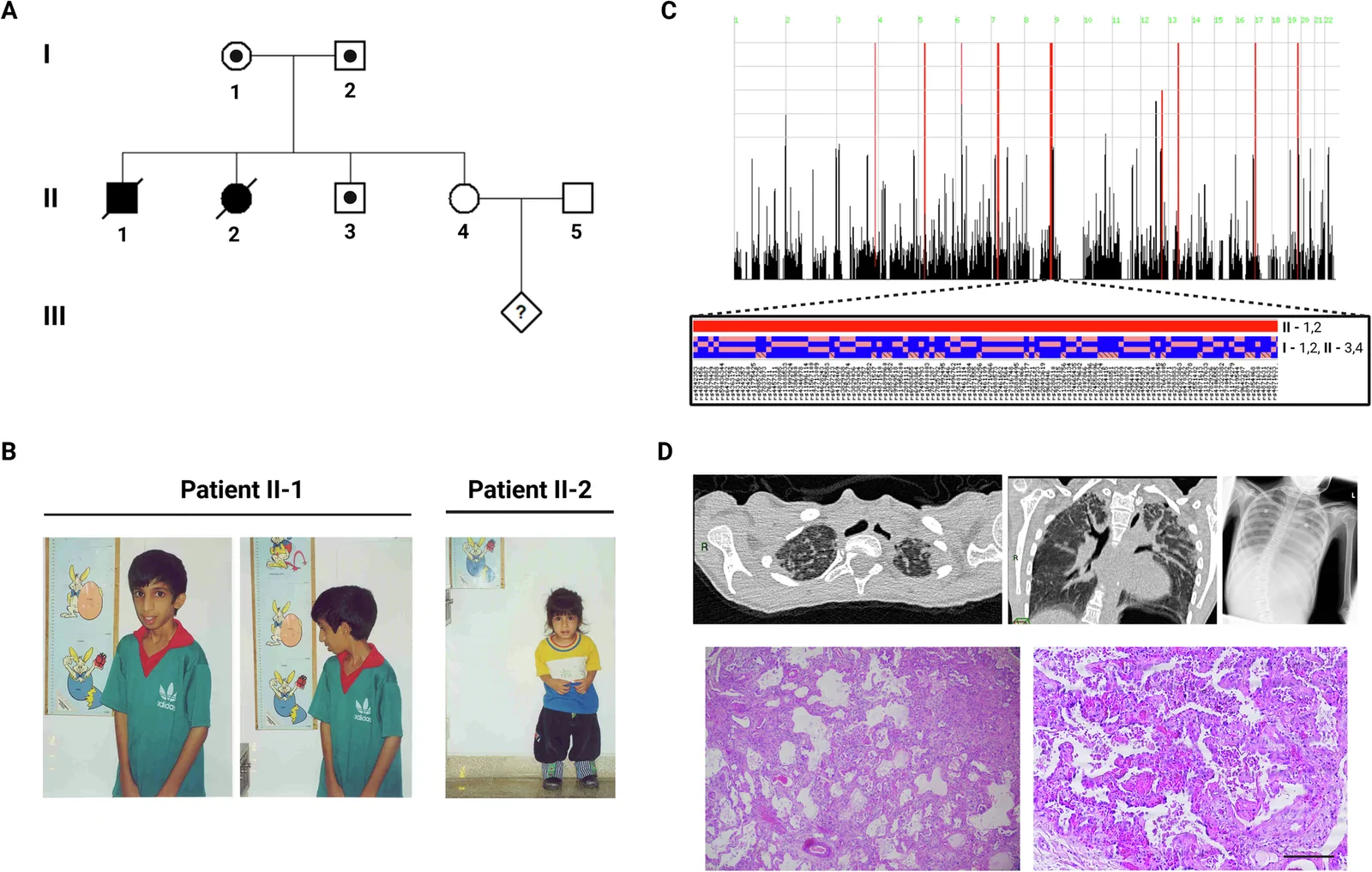
Pedigree, patient’s phenotype, and linkage analysis. **A** Pedigree of a Jewish family of Indian origin. *Black–*affected individuals. *White*–unaffected individuals. *Dots*–obligated carriers **B** Patient II-1 (11 years) and Patient II-2 (3 years), both with height below the 3rd percentile. Dysmorphic features include a thin, elongated face, low-set ears, hypotelorism, and a large nose. **C** Genome-wide homozygosity scores produced by HomozygosityMapper are plotted as a bar chart; *red bars* indicate the most promising homozygosity loci shared only by the affected individuals. *Green numbers* refer to chromosome numbers. Inset box illustrates loci shared between affected and healthy family members within chromosome 8. **D** High-resolution axial and coronal chest CT images demonstrate low lung volumes and upper lobe–predominant fibrosis, characterized by subpleural and peribronchial thickening, reticulation, traction bronchiectasis, and architectural distortion. Frontal chest radiograph shows thoracolumbar scoliosis and diffuse bilateral interstitial opacities. A large air-space consolidation in the left lower lobe is noted, concerning for superimposed pneumonia. Postmortem biopsy from the right lower lobe demonstrates diffuse alveolar damage, with features of both exudative and proliferative phases. Histological findings include alveolar septal thickening, interstitial fibrosis, and prominent hyperplasia of type II pneumocytes, along with focal hyaline membrane formation and intra-alveolar edema.

A family of Indian (Mumbai region) Jewish descent, with no reported consanguinity, presented with two affected siblings - male and female, diagnosed with PD (Fig. 1A). Patient II-2 initially presented with intrauterine growth restriction (IUGR), while other prenatal evaluations were unremarkable. Delivery occurred at 37 weeks of gestation following induction of labor, with a birth weight of 2300 g (consistent with small for gestational age). Postnatally, the patient exhibited severe failure to thrive (FTT) and growth restriction; her height was 74 cm at 3 years of age and 113 cm at 13 years, both remaining below the 1st percentile (Z = −6.0). At 13 years, her weight was 13 kg (Z = −3.2), with a BMI of approximately 10.2 kg/m² and marked paucity of adipose and muscle tissue. Endocrine evaluation of the growth hormone (GH)/IGF-1 axis was performed at two time points and yielded results within normal ranges; At 2.5 years, GH was 5.68 ng/mL and IGF-1 was 10.3 nmol/L. At 11.5 years, an arginine GH stimulation test demonstrated an adequate response, with a peak GH level of 12.9 ng/mL and a normal IGF-1 level (21.5 nmol/L), effectively ruling out growth hormone deficiency.

Throughout her development, the patient experienced recurrent episodes of clinically diagnosed pneumonia, managed with antibiotics and bronchodilators. Over time, continuous prophylactic antibiotic therapy (once-daily Amoxicillin-clavulanate) was initiated. She was considered asthmatic and experienced dyspnea even after minimal exertion. A progressive decline in respiratory function was observed, with pulmonary function testing revealing a severely restrictive lung pattern. Due to concerns regarding chromosomal instability, imaging was initially avoided; however, CT and chest X-ray obtained during her final hospitalization (at age 16) demonstrated findings consistent with severe pulmonary fibrosis. Imaging revealed markedly reduced lung volumes and extensive bilateral interstitial changes, including peripheral interstitial opacities. Significant right-convex scoliosis of the lower thoracic and upper lumbar spine was noted (Fig. 1D). Bronchoscopic evaluation at age 13 demonstrated mild-to-moderate tracheomalacia, characterized by dynamic airway collapse during expiration, while the bronchial mucosa appeared normal. Postmortem biopsy of the right lower lobe (RLL) of the lung in patient II-2 demonstrated diffuse alveolar damage, comprising both exudative and proliferative phases, with a predominance of the proliferative component. Features of the exudative phase included focal interstitial and alveolar edema, focal hyaline membrane formation, vascular congestion with extravasation of erythrocytes, and focal necrosis of alveolar epithelial cells. In parallel, proliferative-phase changes were evident, including marked hyperplasia of type II pneumocytes lining the alveolar surfaces, thickening of alveolar walls, alveolar septal fibrosis, and interstitial fibrosis (Fig. 1D).

Cytogenetic studies revealed marked chromosomal instability, including spontaneous and induced chromosomal breakage and polyploidy (details below). Immunological evaluation at 13 years of age suggested immune dysregulation rather than a definitive immunodeficiency. Flow cytometry demonstrated a mildly reduced CD4⁺ T-cell fraction (28.9%) with a borderline CD4/CD8 ratio ( ~ 1.0), and an increased proportion of CD4⁻/CD8⁻ double-negative T cells (9.4% of total T cells). B-cell analysis revealed an increased total CD19⁺ population (29.6%) but a reduced fraction of memory B cells (CD20⁺CD27⁺: 2.5%), suggesting impaired B-cell maturation. Immunoglobulin profiling showed low IgM (37.9 mg/dL), normal IgG (1330 mg/dL), and low-normal IgA (52.7 mg/dL). Clinically, the patient had one episode of fish-related allergic rash but no other significant allergies. While standard vaccinations yielded appropriate responses, seasonal viral infections followed a prolonged course.

Further endocrinologic evaluation revealed primary hypothyroidism, diagnosed at five years of age, and insulin-dependent diabetes mellitus (IDDM) at fifteen. Thyroid function tests initially showed mildly elevated TSH at 2.5 years (5.38 mIU/L), which normalized by age 3. However, progressive TSH elevation occurred between ages 14.5 and 15 (peaking at 25.13 mIU/L), while FT4 remained within the normal range (0.9 ng/dL). The patient was treated with levothyroxine (Eltroxin), with dose escalation at age 15 resulting in clinical stabilization. Mildly elevated liver enzymes had been noted since birth. Dysmorphic features included a thin, elongated face with hypotelorism, a prominent nose, low-set ears, pectus excavatum, multiple café-au-lait and hypopigmented spots, and hirsutism (Fig. 1B).

Academically, she was a successful student with high grades, attending a mainstream school at the age of 13. No hair loss, oral ulcers, or joint-related symptoms were observed. Cardiac evaluation at 13 years was performed due to prior concerns for pulmonary hypertension. Electrocardiogram demonstrated sinus rhythm with a tendency toward tachycardia, but echocardiography revealed normal cardiac anatomy with preserved systolic function (fractional shortening ~40%). No structural abnormalities, valvular dysfunction, or pericardial effusion were identified. Pulmonary pressures were within normal limits at the time of evaluation (estimated tricuspid regurgitation gradient of approximately 35 mmHg). Mild anemia was present. The patient succumbed at 16 years of age to respiratory failure secondary to interstitial lung disease.

Patient II-1, a male sibling, was born at term with a birth weight of 2600 g. Like his sister, he exhibited severe growth restriction; at 11 years of age, his height was recorded at 124 cm, remaining well below the 1st percentile (Z = −2.7). His dysmorphic features mirrored those of Patient II-2, and he also presented with undescended testes and a systolic heart murmur. Radiographic imaging at age 13 revealed a long, narrow, and small thorax with a loss of physiological kyphosis and central thoracic scoliosis, consistent with straight back syndrome. These skeletal abnormalities resulted in a secondary leftward displacement of the heart.

The patient’s respiratory progression was severe and complex. Longitudinal imaging demonstrated diffuse interstitial lung disease with ground-glass opacities and extensive fibrosis. Findings included bullous emphysema with cystic components in the left upper and lower lobes, alongside cystic bronchiectasis in the right lower and left upper lobes. Tracheobronchomegaly and left-sided pleural thickening were also noted, supporting a diagnosis of advanced and debilitating pulmonary disease. Complicating his clinical course, the patient suffered a severe episode of varicella (chickenpox), further suggesting underlying immune dysregulation.

Endocrine evaluations revealed fluctuating thyroid function. At age 13, TSH was elevated (17.74 uIU/mL) with a normal FT4, followed by a period of normalization. By age 14, the patient exhibited a suppressed TSH (0.08 uIU/mL) with an elevated FT4 (2.1 ng/dL) and low FT3 (1.6 pg/mL), which subsequently shifted back to an elevated TSH (7.48 uIU/mL) within a week. At 13.5 years of age, the patient succumbed to complications of pneumonia and pneumothorax, following a course of progressive pulmonary deterioration similar to that of his sibling.

There are no additional affected family members. Of note, their maternal grandfather was diagnosed with asthma, their father (I-2) died of a myocardial infarction, and two other siblings (II-3 and II-4) are healthy.

### Cytogenetic studies

While standard karyotype and CMA were normal, cytogenetic analysis of PHA-stimulated lymphocytes demonstrated in both affected individuals chromosomal fragility and polyploidy: individual II-1 (male) presented 17–35% polyploidy and individual II-2 (female), 35–70%. In both cases, there was no further effect when cells were exposed to DNA cross-linking agents DEB or MMC.

### Molecular genetic analysis

Initial laboratory tests of the affected individuals, including standard peripheral lymphocyte karyotype and chromosomal microarray (CMA), were normal. Sanger sequencing of *FANCA* was normal - ruling out a p.S858R *FANCA* mutation previously described in Jews of Indian descent [14], and no mutations were found in Nibrin P95 [15].

Six family members underwent linkage analysis: the parents (Fig. 1A; I-1,I-2) marked as obligatory carriers, the two affected siblings (II-1, II-2), and two healthy siblings (II-3, II-4). Assuming autosomal recessive heredity of the disorder, HomozygosityMapper identified 7 possible disease-associated genomic loci (Manhattan plot, Fig. 1C) spanning a total of ~17.5Mbp: Chr3: rs9837491-rs9839753, Chr5: rs29738 - rs13176570, Chr8: rs13248065 - rs4871014, Chr12: rs2247919 - rs12296211, Chr13: rs2296122 - rs9535091, Chr17: rs4790733 - rs8064677, Chr19: rs10418827 - rs10426628 (Fig. 1C).

**Fig. 2:**
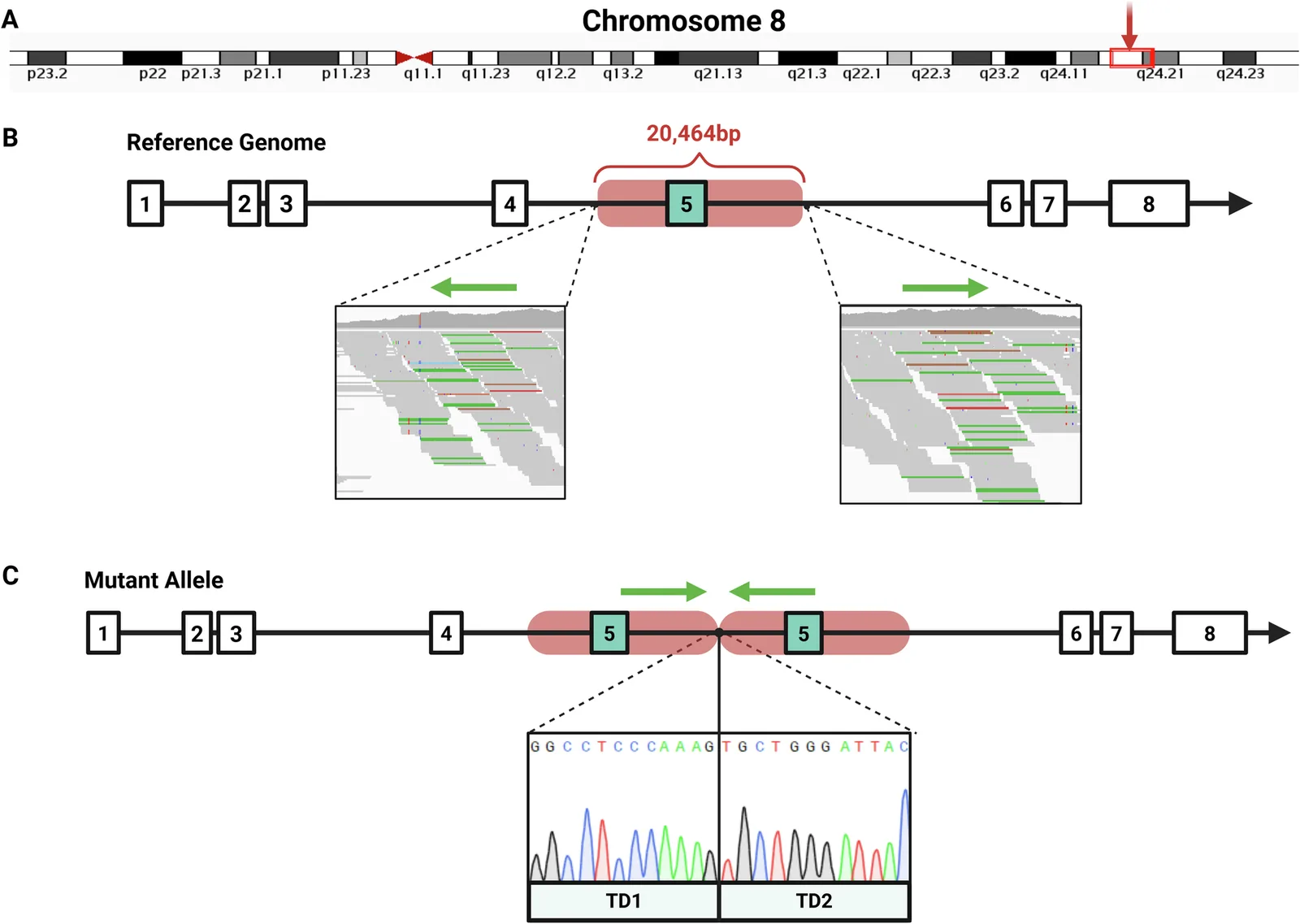
*NSMCE2* Structural variant identification and verification. **A** Schematic representation of chromosome 8. The red box indicates the linkage region (8q24.13–q24.21), and the red arrow marks the position of the *NSMCE2* gene within this interval. **B** IGV visualization of short-read sequencing data aligned to the reference genome at the *NSMCE2* locus. *Red shaded boxes* denote the 20,464 bp duplication encompassing exon 5. Green arrows indicate discordant paired-end reads supporting the duplication event. **C** Schematic illustration of the predicted tandem duplication in *NSMCE2*. Green arrows represent the orientation of paired-end reads flanking the breakpoint junction in the mutant allele. Sanger sequencing of PCR-amplified products confirmed the precise breakpoint junction between the duplicated segments.

Whole exome sequencing (WES) studies of individual II-1 using both VARISTA [10] analysis tool and Genoox analysis software “Franklin” failed to identify likely disease-causing variants within the above loci or in any of the genes previously associated with chromosomal breakage syndromes or primordial dwarfism. Whole genome sequencing (WGS) for individual II-2, exploring non-coding regions, did not reveal any relevant SNVs in the disease-associated loci. However, Manta software analysis of the WGS data for structural variants (SV) identified a tandem duplication (TD): a structural rearrangement in which a DNA segment is duplicated and inserted serially alongside the original segment. The TD identified (NC_000008.11:g.125170672_125191135dup) within the chromosome 8 disease-associated locus, spans 20,464 bp, within the *NSMCE2* gene (Fig. 2A). The TD encompasses exon number 5 of *NSMCE2* (Fig. 2B), as well as extensive intronic regions (NC_000008.11(NM_173685.4):c.265-11431_418+8879dup) which are predicted to contain six distinct enhancer elements [16]. Screening of 43 ethnically matched controls revealed no carriers or homozygotes for this TD (Fig. S1). The variant was also absent from the Genome Aggregation Database for Structural Variants (gnomAD SVs v4.1.0) [17], which includes data from 63,046 genomes, as well as from dbVAR14 [18]. Moreover, the gnomAD database for single-nucleotide variants (SNVs) shows no homozygous individuals with predicted pathogenic loss-of-function variants among healthy individuals.

The SV (NC_000008.11:g.125170672_125191135dup) was classified as pathogenic according to the ACMG/AMP and CNV classification guidelines. Variant scoring is provided in Supplementary Table 1.

Our finding was validated by visualizing the borders of the TD on the BAM file using the Integrative Genomics Viewer (IGV) [19], noting the presence of 22 discordant read pairs (Fig. 2B). We successfully PCR-amplified the region at the junction site between the two duplicated segments. Amplification was observed exclusively in individuals carrying the mutant allele, whether in the biallelic or monoallelic state. The PCR results were consistent with the predicted zygosity of the family members based on SNP-array linkage analysis: amplification was detected in biallelic affected individuals (II-1 and II-2) and in monoallelic carriers (I-1, I-2, and II-3). No PCR amplification was detected in the healthy family members (II-4 and II-5), who do not carry the mutant allele (Fig. S1). Sanger sequencing of the PCR amplicons confirmed the precise junction point between the two duplicated regions (Fig. 2C).

**Fig. 3:**
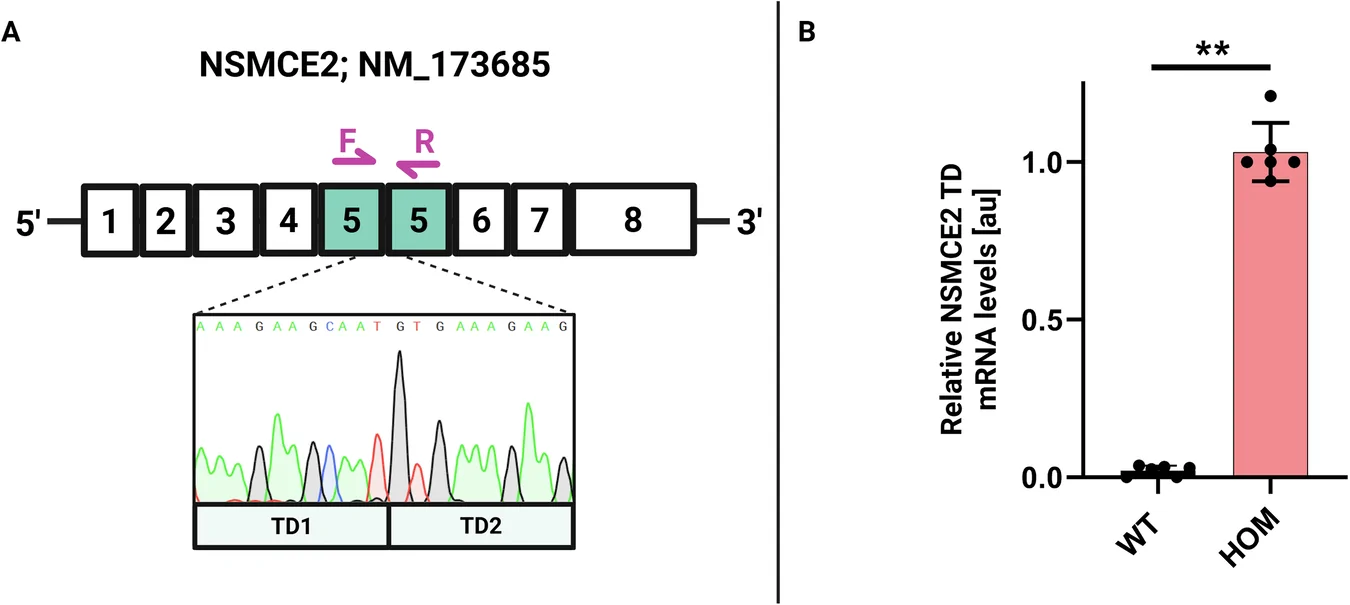
Molecular analysis of the *NSMCE2* structural variant impact. **A** Mutated *NSMCE2* cDNA illustration (NM_173685). *Green squares* indicated the duplicated exon 5. *Purple arrows* indicate primers forward (F) and reverse (R), which flank the junction point between the two duplicated exon 5. Sanger sequencing confirmed the junction point. **B** RT-PCR quantification of the aberrant transcript: Homozygous affected individuals (II-1, II-2) compared to a homozygous WT control. Each dot represents a measurement. Data were analyzed using a two-tailed Mann-Whitney test; *P* = 0.0022.

We hypothesized that the mutation would disrupt splicing, leading to the generation of an aberrant transcript containing a tandem duplication of exon 5. To test this, we generated cDNA from EBV-transformed lymphoblastoid cells of affected individuals and controls, followed by PCR amplification using primers flanking the predicted exon 5 duplication junction. Sanger sequencing confirmed the presence of the aberrant transcript (Fig. 3A). Real-time PCR analysis further demonstrated that the *NSMCE2* aberrant transcript, spanning the duplication junction, was virtually undetectable in WT control samples compared to a homozygous affected sample (Fig. 3B).

## Discussion

We further describe an autosomal recessive syndrome characterized by IUGR followed by FTT, postnatal growth restriction / primordial dwarfism, as well as hypotonia, kyphoscoliosis, hirsutism, and multiple café-au-lait and hypopigmented spots. Distinctive dysmorphic features included a thin, elongated face, low-set ears, hypotelorism, and a prominent nose. Hypothyroidism emerged during childhood, and IDDM in adolescence. A major phenotypic hallmark of the syndrome was interstitial lung disease, presenting as severe pulmonary fibrosis with bronchiectasis and recurrent pneumonia, ultimately culminating in death during the adolescent years. There was no evidence of malignancy. While standard karyotype and CMA were normal, cytogenetic analysis of PHA-stimulated lymphocytes demonstrated chromosomal instability and polyploidy, with no further effect induced by cross-linking agents DEB or MMC. WES and WGS failed to identify a disease-causing mutation. However, combining linkage analysis with Manta software analysis of WGS data, using a previously described approach [20], we demonstrated that the disease is caused by a biallelic homozygous tandem duplication structural variant (TD-SV) within *NSMCE2*.

To better understand the molecular basis of the syndrome and the impact of the identified *NSMCE2* structural variant, it is essential to first delineate the biological role of Structural Maintenance of Chromosomes (SMC) complexes. SMCs are crucial players in the organization and dynamics of chromosome structures across all living organisms [5]. These complexes comprise three distinct entities: Cohesin (SMC1/3), which acts in tethering sister chromatids together; Condensin (SMC2/4), which facilitates chromosome compaction and elasticity during mitosis; and the Smc5/6 complex, which is involved in DNA repair and maintenance of genomic stability [5]. A human SMC5/6 complex is composed of 2 core SMC components (SMC5 and SMC6) and 4 non-SMC elements (NSE1-4). The non-SMC element number 2 (NSE2), also known as *NSMCE2*, has a major role in maintaining genome stability [21, 22].

**Fig. 4:**
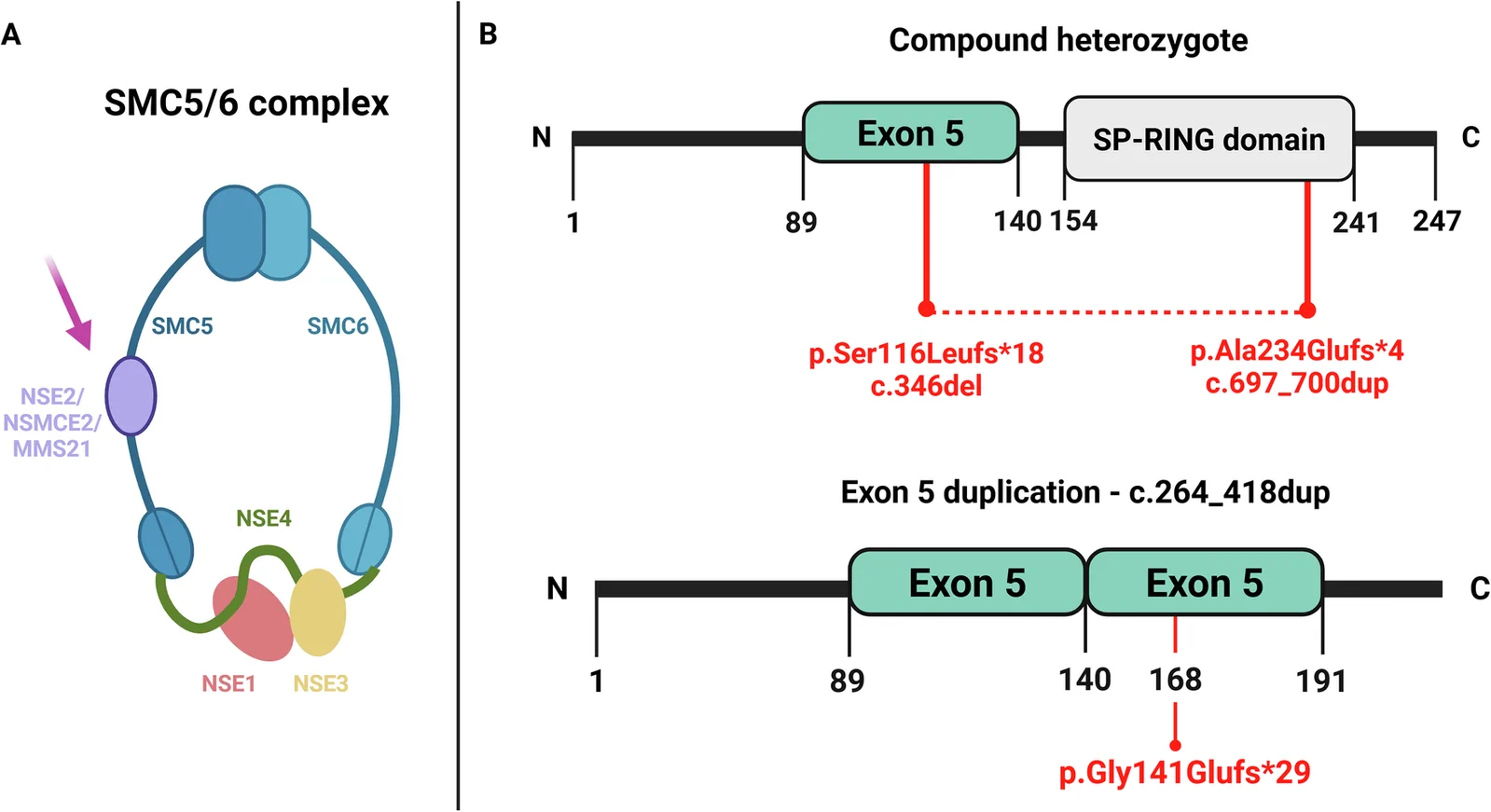
Structural organization of the SMC5/6 complex and *NSMCE2* Mutation overview. **A** Schematic representation of the architecture of the SMC5/6 complex: classic SMC dimer V-shape with the SMC5 and SMC6 proteins interacting at the hinge region. The NSE4 protein bridges the gap between the head domains, and it directly binds to both NSE1 and NSE3. *NSMCE2’s* location within the coiled segment of SMC5. **B** Visualization of *NSMCE2* cDNA (NM_173685) featuring the locations of reported mutations.

*NSMCE2*, also known as *MMS21* (Methyl Methane Sulfonate 21), was initially identified in a screen for mutants sensitive to the alkylating agent methyl methane sulfonate (MMS). It is a conserved component of the SMC5/6 complex across all known organisms [23]. *NSMCE2* docks directly to the coiled-coil region of the SMC5 protein, a configuration distinct from other NSE components (Fig. 4A). Its N-terminal mediates SMC5 attachment, while its C-terminal SP-RING domain confers SUMO E3 ligase activity. This structural arrangement enables NSMCE2 to remain anchored to SMC5 while interacting with SUMO substrates, contributing to the DNA damage response [21–24]. It is thus assumed that mutations abrogating the role of NSMCE2 in recovery from DNA damage culminate in dwarfism through reduced tolerance of replicative stress [25].

As indicated by the Human Gene Mutation Database (HGMD) [26], only a single previous report [25] described two independent cases of primordial dwarfism with mild spontaneous chromosomal breakage due to compound heterozygous mutations in *NSMCE2*. The two previously described affected individuals had primordial dwarfism, paucity of adipose tissue and muscle, gonadal failure, extreme insulin resistance, fatty liver, and hypertriglyceridemia developing in childhood, as well as acanthosis nigricans in lateral neck, axillae and antecubital fossa, with dysmorphic features of prominent midface and small lower jaw. There was no evidence of malignancies or of bone marrow failure. Initial karyotypes were normal, but one of the individuals demonstrated chromosomal breakage on careful cytogenetic analysis. Evidence of chromosome damage lessened with maturity, and by the age of 16.9 years, no excess chromosome damage could be detected. One of the two affected individuals was congenitally blind, reportedly due to bilateral retinal detachment, and died suddenly at age 33 years of a sudden cardiovascular event.

The phenotype observed in the two patients we describe overlaps with that of the two previously reported cases [25]: primordial dwarfism, paucity of adipose tissue and muscle, and chromosomal breakage were evident in all four individuals. None of the patients showed evidence of bone marrow failure or malignancy. Notably, the dysmorphic features described in the previously published cases closely resemble those seen in our patients, including a thin, elongated face, low-set ears, and a prominent nose. However, the clinical presentation in our patients was significantly more severe and expanded, ultimately affecting life expectancy. This was reflected by markedly increased chromosomal breakage and the presence of interstitial lung disease with severe pulmonary fibrosis, bronchiectasis, and recurrent pneumonia, culminating in earlier death at the ages of 13 and 16 years. Additional features identified in our patients included hypotonia, kyphoscoliosis, hirsutism, and multiple café-au-lait and hypopigmented spots.

The milder clinical presentation in the two previously reported cases may be explained by the nature of the compound heterozygous mutations, which likely allow residual stability and functionality of the SMC5/6 complex. The two previously reported cases, although not formally related, had shared compound heterozygous mutations (Fig.4B); NM_173685.4:c.346del, c.697_700dup, NP_775956.1:p.(Ser116Leufs*18), p.(Ala234Glufs*4) [25]. The findings regarding loss of function due to the mutations were indicative of possible residual activity: *NSMCE2* mRNA levels in dermal fibroblasts of affected individuals were 50% of WT levels, while in lymphoblast cell lines they were near normal. Furthermore, patients’ lymphoblasts displayed mild under-expression of SMC5 and SMC6 proteins, indicating only modest instability of the core SMC5-6 protein complex. Notably, in heterologous protein overexpression experiments using a Myc-tagged version of *NSMCE2* carrying one of those previously-reported mutations (NP_775956.1:p.Ala234Glufs*4) the mutant NSMCE2 protein was detected on immunoblotting at levels close to those in cells overexpressing the WT protein [25].

The homozygous ~20.5 kb tandem duplication (TD) identified in our patients, encompassing the entire exon 5 of *NSMCE2*, is likely more disruptive than the previously reported mutations and may account for the more severe phenotype observed in our patients. This novel TD-SV generates an aberrant *NSMCE2* transcript with a full duplication of exon 5 (154 bp for each copy), which is predicted to introduce a deleterious frameshift at amino acid 141, ultimately leading to a premature stop codon at amino acid 168. (NP_775956.1:p.Gly141Glufs*29; Fig. 4C). This abnormal transcript is expected to trigger nonsense-mediated decay (NMD), resulting in reduced NSMCE2 protein levels. Because the effect of the duplication depends on splicing, the spliceosome may occasionally skip one of the duplicated exons during splicing and generate correctly spliced transcripts; therefore, some residual NSMCE2 protein expression and activity in affected individuals are plausible. However, direct experimental evaluation of this predicted protein effect was not feasible due to limitations of the available patient-derived material. Patient-derived lymphoblastoid cell lines were generated more than two decades ago and had been stored at −80 °C rather than under standard cryopreservation conditions in liquid nitrogen. Attempts to revive these archival cells were unsuccessful. Nevertheless, some RNA could be extracted from the stored material, allowing PCR amplification across the duplication junction and demonstrating the presence of the aberrant transcript. These findings support the predicted transcript consequence of the tandem duplication.

The resulting premature termination codon lies upstream of the Siz/PIAS RING (SP-RING) domain, which is located at the C-terminus and confers SUMO E3 ligase activity. In contrast, previously reported compound mutations included a variant that did not completely disrupt the activity of the SP-RING domain (Fig. 4B). While it has been previously suggested that the SUMO ligase activity of NSMCE2 is dispensable for its core functions, such as chromosome segregation and genome maintenance [27], our findings support a significant functional role for this activity. The wide phenotype we delineate, with primordial dwarfism, immune deficiency, lung abnormalities, gonadal failure and late-onset insulin resistance, implies that the SUMO ligase function of NSMCE2 may play a crucial role in DNA damage repair in a broad array of mammalian tissues.

A key feature of nearly all cancers is genome instability, which allows for enhanced somatic genetic modifications during cell growth and division, with high levels of replication stress, driving cancer initiation. Given NSMCE2’s role in replication stress recovery and DNA damage repair, one might expect loss-of-function mutations to predispose to malignancy [23]. However, no malignancies were reported in our patients or in the previously described cases, possibly due to early childhood mortality masking long-term risk.

Notably, a disease phenotype similar to the *NSMCE2* deficiency syndrome, with defects in DNA repair and chromosomal instability, was recently described in adult patients with a homozygous in-frame deletion of a specific amino acid within the *SMC5* gene (NM_015110.4:c.1113_1115del, NP_055925.2:p.Arg372del). The deleted amino acid was shown to disrupt the SMC5 interaction site with the NSMCE2 protein [28]. Intriguingly, the identification of biallelic missense mutations in *NSMCE3*, (NM_138704.3:c.790 C > T, NP_619649.1:p.Leu264Phe and NM_138704.4:c.626 C > T, p.Pro209Leu), another component of the SMC5/6 complex, has revealed overlapping yet also markedly different and more severe clinical features. These mutations caused a chromosome breakage syndrome associated with severe pulmonary disease in early childhood. The rationale behind this observation lies in the crucial role of the *NSMCE3* subunit in maintaining the structural integrity of the SMC5/6 complex; its absence destabilizes the entire complex. Hence, the authors hypothesized that the observed effects result from the loss of the entire functions of the complex, rather than being solely attributable to the absence of *NSMCE3*. This speculation is supported by the fact that *NSMCE3* levels in their patients’ fibroblasts were below the level of detection, accompanied by reduced levels of other proteins such as SMC5 and SMC6 [29]. Our research suggests that biallelic *NSMCE2* variants predicted to substantially reduce *NSMCE2* function may result in a syndrome of comparable severity to that observed in *NSMCE3* mutations, which impair the integrity of the entire SMC5/6 complex. However, because direct protein studies were not feasible, and residual correctly spliced *NSMCE2* transcripts may still be generated, complete loss of *NSMCE2* function could not be established in our patients.

Our findings underscore the importance of screening for SVs when conventional sequencing methods fail to identify causative SNVs, indels, or chromosomal anomalies. Standard WES and WGS did not detect the TD in our study. SVs may not be identifiable via WES when their boundaries lie within an intronic or intergenic region. The integration of WGS with Manta software was critical in identifying this variant, as previously described [20]. It is noteworthy that specifically homozygous TDs can pose complexities in detection and interpretation using IGV screening tools, as, unlike various other structural variants, TDs may exhibit a “heterozygous appearance”. Approximately 50% of the reads can align normally at the duplicated segment borders, which is attributed to their presence also in the mutated allele, as evident in our case (Fig. 2A) and illustrated in Fig. S2.

Studies in murine models have demonstrated that complete loss of *NSMCE2* is embryonically lethal, with homozygous knockout embryos failing to develop beyond early pre-implantation stages due to severe chromosome segregation defects. These defects were characterized by abnormal nuclear morphology and mitotic failure. In contrast, mice harboring SUMO-ligase-dead mutations in *NSMCE2*, which disrupt the SP-RING domain, remain viable and fertile, suggesting that SUMOylation activity is largely dispensable for development and normal lifespan under unstressed conditions. Conditional *NSMCE2* deletion in adult mice, however, leads to progressive progeroid features, including growth restriction, altered pigmentation, anemia, and accumulation of micronuclei, mimicking features of Bloom syndrome. The fact that the SV identified in our patients is not embryonically lethal in humans supports the hypothesis that some residual NSMCE2 protein expression might have persisted, potentially due to occasional skipping of one duplicated exon during splicing.

Moreover, *NSMCE2*-deficient cells exhibit increased mitotic recombination, elevated telomeric SCE, and segregation errors, highlighting the essential role of *NSMCE2* in genome stability, recombination suppression, and proper chromosome segregation, independent of its SUMO E3 ligase activity [23, 27].

In summary, we have validated and significantly broadened the phenotype associated with the *NSMCE2* mutation syndrome, most notably with immune deficiency and severe lung disease, and have provided a comprehensive overview of all known pathogenic mutations within the SMC5/6 complex to date.

## Supplementary information

Supplementary Figure S1

Supplementary Figure S2

Supplementary Table I: ACMG Classification

## Data Availability

Data beyond those given in the manuscript are available from the corresponding author upon reasonable request. The data are not publicly available because they contain information that could compromise research participant privacy/consent.
