# Supplementary material for: NSMCE2 / MMS21 structural variant causes chromosome breakage syndrome with primordial dwarfism and severe lung disease
Source: Eur J Hum Genet. 2026 Sep 21;34(11):1685–92. doi: 10.1038/s41431-026-02222-7 (PMC13743549; doi:10.1038/s41431-026-02222-7)

Supplementary Figure S1: (A) PCR across the junction between the two duplicated regions identified carriers of the structural variant within the family. Amplification was detected in affected biallelic individuals (II-1 and II-2) and in heterozygous unaffected family members (I-1, I-2, and II-3), but not in healthy relatives lacking the variant (II-4 and II-5). **(B) Screening of 43 ethnically matched Indian control individuals for the identified mutation.** PCR and Sanger sequencing revealed the absence of the variant in all control samples. PC, Positive control.

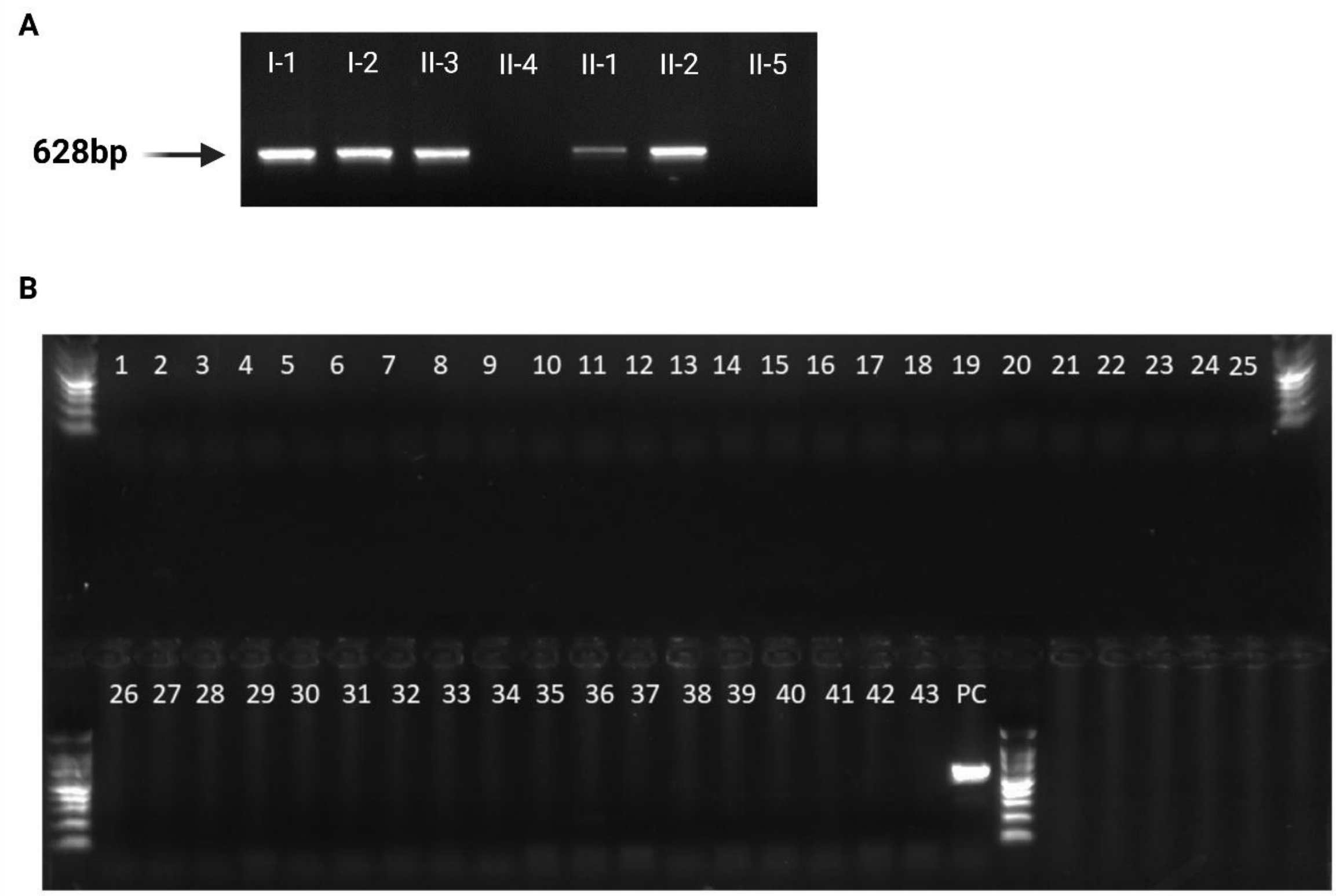

Supplement: Supplementary file 1 [file 41431_2026_2222_MOESM1_ESM.pdf]
