# Supplementary material for: NSMCE2 / MMS21 structural variant causes chromosome breakage syndrome with primordial dwarfism and severe lung disease
Source: Eur J Hum Genet. 2026 Sep 21;34(11):1685–92. doi: 10.1038/s41431-026-02222-7 (PMC13743549; doi:10.1038/s41431-026-02222-7)

Supplementary Figure S2: Illustration of tandem duplication (TD) paired reads alignment on mutant and WT references. *Grey arrows*; Paired reads that demonstrate proficient alignment with the duplicated region borders on both the wild-type and mutated reference sequences. *Green arrows;* Paired reads exclusive to the mutated genome, efficiently aligning with the mutated reference, but when mapped to the wild-type (WT) reference, they display an anomalous orientation, causing discordance.

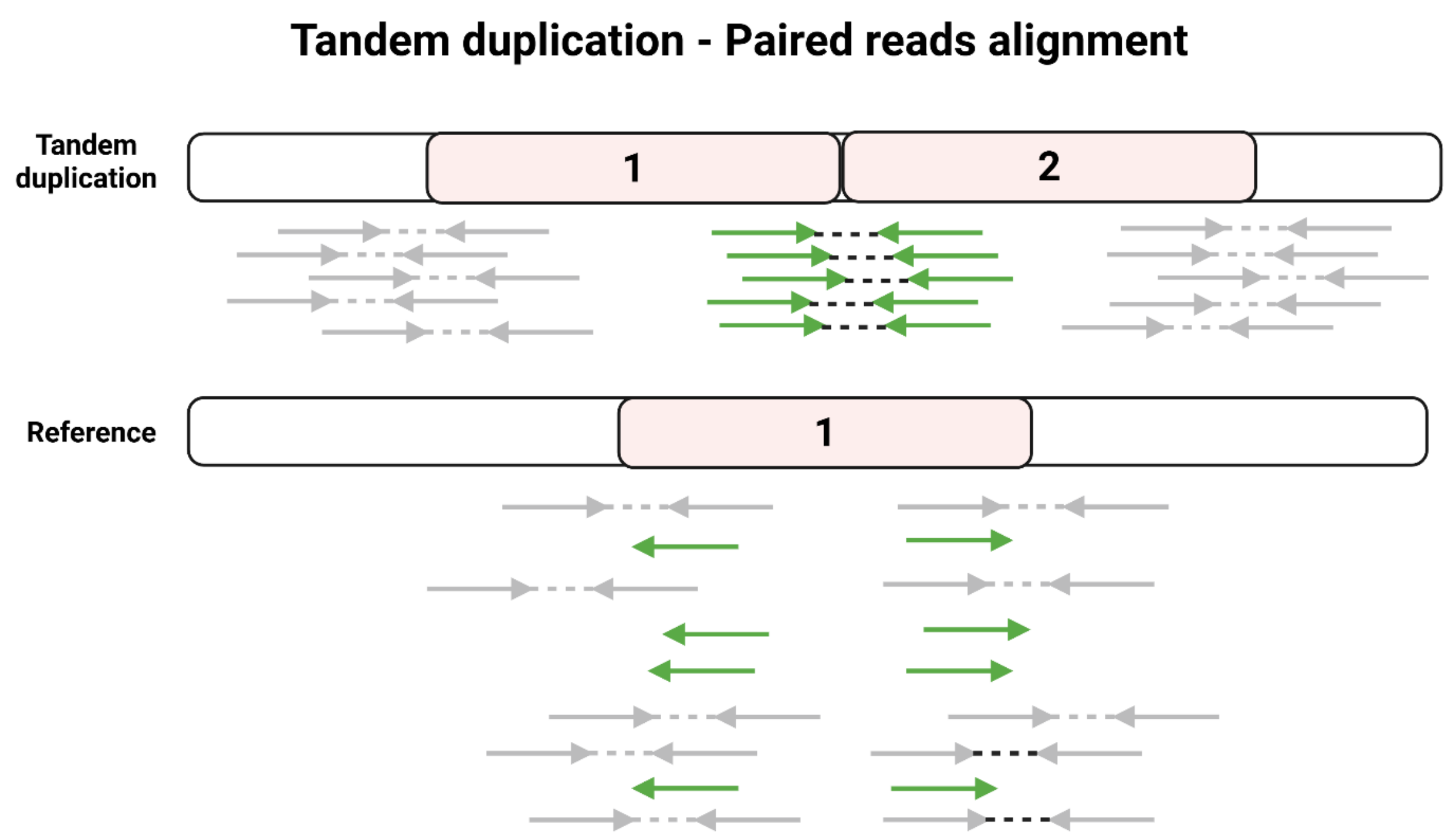

Supplement: Supplementary file 2 [file 41431_2026_2222_MOESM2_ESM.pdf]
